# Harnessing bacterial power and omics technologies for sustainable plastic waste biodegradation

**DOI:** 10.1007/s10532-026-10258-1

**Published:** 2026-03-07

**Authors:** Ahmed R. Henawy, Salma M. Ismail, Sama Gharib, Nagwa I. Elarabi, Abdelhadi A. Abdelhadi, Asmaa A. Halema

**Affiliations:** 1 Department of Microbiology, Faculty of Agriculture, Cairo University, Giza, 12613 Egypt; 2 Biotechnology Program, Faculty of Agriculture, Cairo University, Giza, 12613 Egypt; 3 Department of Genetics, Faculty of Agriculture, Cairo University, Giza, 12613 Egypt

**Keywords:** Plastic biodegradation, Microbial enzymes, PETase, Omics technologies, Synthetic polymers

## Abstract

Plastic pollution constitutes a critical environmental concern of this era, with synthetic polymers, i.e., polyethylene (PE), polyethylene terephthalate (PET), polystyrene (PS), and polyurethane (PU), accumulating in terrestrial and aquatic ecosystems at alarming rates. One of the promising solutions to this worldwide problem is microbial plastic degradation, particularly by bacteria that can convert polymeric materials into less toxic compounds. With an emphasis on enzymatic mechanisms, critical environmental and biochemical factors influencing degradation, and the wide variety of bacteria responsible for breaking down synthetic polymers, this review focuses on the enzymatic and genetic aspects underlying bacterial plastic degradation, highlighting key enzymes such as PETase, METase, esterase, and oxidoreductase, as well as representative plastic-degrading bacteria i.e*. Thermobifida, Ideonella, Bacillus, Agromyces, Pseudomonas, Schlegelella* species. The significance of multi-omics tools, such as transcriptomics, proteomics, metabolomics, and genomics was demonstrated here in deepening our understanding of microbial plastic degradation without depending on pure culture. It explores the key genes and metabolic pathways that facilitate this process. Moreover, how advanced biotechnological techniques and artificial intelligence (AI) can participate in plastic biodegradation through enzyme engineering, activity-enhancing mutation design, predictive modeling, and omics data analysis was illustrated. Furthermore, this review underscores the necessity for integrative and interdisciplinary approaches to effectively harness bacterial metabolism for long-term reduction of plastic pollution. Also, it outlines future research directions and technological priorities for translating bacterial plastic degradation into practical and sustainable remediation solutions.

## Introduction

Plastic pollution stands among the most urgent environmental issues faced today, stemming from the overproduction, excessive consumption, and improper disposal of plastic materials (Moharir and Kumar 2019). They are synthetic polymers which are valued for their strength, lightweight nature, and versatility. However, they resist the degradation and pose a long-term threat in the environment leading to serious health risks (Gaur et al. 2023, 2025; Kibria et al. 2023).

Microplastics in soil can alter soil structure, impair root development, and reduce nutrient uptake, ultimately affecting plant health and crop productivity (Hasan and Tarannum 2025). Also, marine ecosystems are particularly vulnerable, with plastics damaging coral reefs and affecting the survival of countless aquatic species (Nath et al. 2023).

**Fig. 1:**
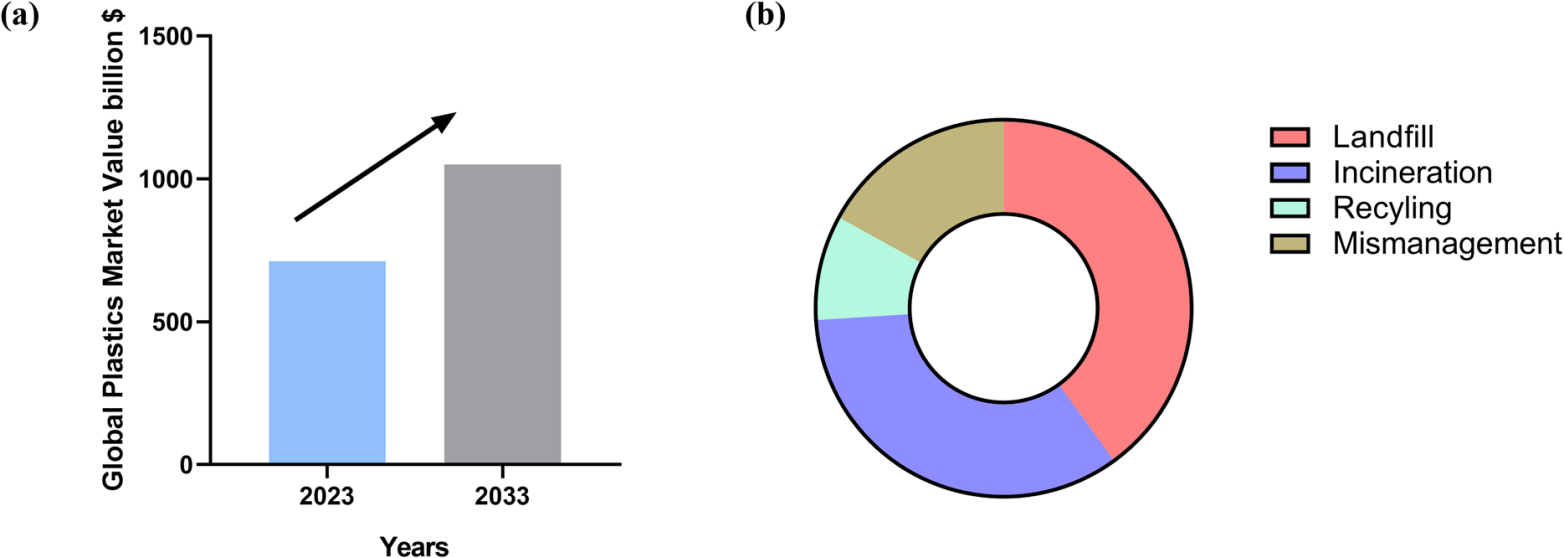
Recent plastic pollution statistics, illustrating the urgency of plastic waste management. **a** global plastics market value, highlighting the rapid growth of plastic production and consumption worldwide, and **b** distribution of plastic disposable pathways percentage, demonstrating the predominance of landfill and incineration over recycling and mismanagement

The scale of plastic pollution continues to grow. In 2023, global plastic production reached 413.8 million metric tons, a figure that underscores the ongoing expansion of this material category, largely attributable to its versatility (Stipp 2024a). The valuation of the global plastics market in 2023 was approximately 712 billion U.S. dollars, with projections indicating an increase to over 1,050 billion U.S. dollars by 2033 (Stipp 2024b) as illustrated in Fig. 1a.

Annually, millions of tons of plastic waste are introduced into natural ecosystems, particularly affecting oceans, rivers, and forests. This waste ranges from large, visible items to microscopic fragments termed microplastics, which permeate soil, aquatic environments, and even the atmosphere (Cai et al. 2023). Such pollution has detrimental effects on wildlife through ingestion and entanglement, disrupts ecosystems, contributes to climate change, and poses significant health risks to humans. It is estimated that around eight to ten million tons of plastics enter the ocean yearly, encompassing both macroplastics and microplastics, constituting 80% of all marine pollution (Pilapitiya and Ratnayake 2024). Plastic waste is responsible for the mortality of more than one hundred thousand marine species annually, affecting habitats ranging from coastal regions to the deep ocean (Cózar et al. 2014; Van Sebille et al. 2015).

Several polymeric materials i.e., polyethylene (PE), polystyrene (PS), polypropylene (PP), polyvinyl chloride (PVC), polyethylene terephthalate (PET), polyurethane (PUR), polycaprolactone (PCL), and others, are highly resistant to degradation. These materials consequently gradually build up in the environment, resulting in a serious environmental problem that demands the immediate development of novel treatment and control methods (Yang et al. 2023). Additionally, Houssini et al. (2025) reported that there has been a notable change in how waste is disposed through landfills are declining significantly (40%), incineration is becoming a popular technique (34%), and the global recycling rate has remained the same (9%), as shown in Fig. 1b. Only small amounts of plastic waste are successfully recycled, despite ongoing efforts to enhance recycling and waste management. Unfortunately, these conventional plastic waste management practices face significant limitations. Plastics are capable of degradation in natural environments by mechanical, photochemical, thermal, and biochemical processes (Gewert et al. 2015). Mechanical recycling is the most common method, involving sorting, cleaning, melting, and remolding plastic into new products. While it is cost-effective and helps reducing the demand for new plastic, it can only be applied to certain types of clean plastics and results in lower quality products over time (Vollmer et al. 2020). Chemical recycling breaks plastics down into their original chemical components through processes like pyrolysis or depolymerization (Rahimi and García 2017). Although it can handle mixed or contaminated plastics and recover valuable resources, it is expensive, energy-intensive, and often polluting (Alaghemandi 2024). Incineration, a process that involves the combustion of plastics to diminish their volume and occasionally generate energy, is effective in waste reduction; however, it emits harmful pollutants and contributes to climate change (Shen et al. 2020). Landfilling, a commonly employed yet unsustainable practice, entails the deposition of plastics in terrestrial environments, where they may persist for centuries, leach toxic substances, and occupy valuable land resources. In contrast, microbial degradation represents an emerging, environmentally sustainable approach that utilizes bacteria and fungi to decompose plastics into innocuous byproducts such as carbon dioxide and water (Wojnowska-Baryła et al. 2022).

Hence, reducing the pollution of plastic needs a comprehensive strategy which includes improving waste management, reducing plastic production, and calling for alternatives to single use plastics (Fayshal 2024). Microorganisms have demonstrated efficacy in degrading specific types of plastics. This biological method operates under mild conditions and presents a sustainable solution; however, it remains in the nascent stages of development and faces challenges concerning efficiency, scalability, and a diverse array of plastic materials (Jadaun et al. 2022). Microbial degradation holds significant promise for the future of plastic waste management, providing a cleaner and more natural alternative to traditional methods (Oliveira et al. 2020).By microbes, natural or synthetic plastics can be degraded(Alshehrei 2017; Sutaoney et al. 2025).

These microorganisms utilize complex enzyme systems to efficiently break down plastic polymers and extract energy from them (Chen et al. 2020). However, microbial degradation occurs at a very slow rate, mainly serving to release energy for cellular growth (Crawford and Quinn 2017; Zumstein et al. 2018).

Advances in omics-based technologies have enabled the rapid identification of novel genes, enzymes, and pathways involved in bioremediation without the need to isolate microbes. These technological developments, particularly the rapid progress in next-generation sequencing, have significantly enhanced our understanding of the dynamic composition of microbial communities in ecosystems affected by plastic (De Tender et al. 2015; Malik et al. 2023).

Despite the vast reviews addressing plastic biodegradation, most existing reviews focus on traditional waste management approaches or demonstrate a summary of plastic-degrading microbes. A comprehensive insight which integrates bacterial plastic biodegradation with cutting edges in multi-omics remains limited. This review uniquely fills this gap. This review highlights the significance of plastic-degrading bacteria, the key genes linked to this process, and the role of multi-omics techniques in uncovering these mechanisms.

## Biochemistry of plastic degradation

### Mechanism of plastic biodegradation

Monomers polymerize into macromolecular chains to form plastic polymers. Polymerization needs several materials and monomers i.e., initiators, catalysts, and solvents, relying on the production method (Lithner et al. 2011). Plastic biodegradation can be primarily performed through the depolymerization of polymer chains by enzymes, which produces intermediates with modified characteristics that make them easier for cells to absorb (Zhang et al. 2020). Three separate processes are involved in the polymers biodegradation: (i) microorganisms adhering to the polymer’s surface, (ii) using the polymer as a source of carbon, and (iii) the polymer’s ultimate breakdown.

#### Microorganisms adhering to the polymer’s surface

According to Danso et al. (2019), microbes adhere themselves to the polymer surfaces and aid in their breakdown by releasing enzymes that are used to generate growth energy. Also, this process ultimately breaks down large polymers into monomers and oligomers. Certain oligomers can diffuse within microorganisms and then be assimilated in their internal milieu.

#### Biodeterioration of polymer surface

Assimilation, mineralization, bio-fragmentation, and biodeterioration are all systematic mechanisms of microbial polymer degradation. The process that modifies plastics' surface and alters their chemical, mechanical, and physical characteristics is called biodeterioration. All structural and chemical alterations rely on the content and structure of polymers. The environment has an impact on how the characteristics of polymers vary as well. According to Oliveira et al. (2020), the process of deterioration is the cause of both biofilm production and substrate formation inside the plastic.

#### Bio-fragmentation and assimilation

Following biodegradation, plastic polymers undergo enzymatic action to undergo bio-fragmentation. Since oxygenases, which are primarily bacterial enzymes, can add oxygen to carbon chains and result in their breakdown, less hazardous alcohol and peroxyl compounds are produced (Pathak and Navneet 2017). Additionally, endopeptidases for amide groups or lipases and esterases catalyze the transition of carboxylic groups. During the bio-fragmentation, the resulting plastic polymers pass through the microbial cells. Because of their molecular size, large monomers remain outside the cells without being absorbed. Energy is produced through the oxidation of the tiny monomers that have migrated inside the cells.

#### Mineralization

**Fig. 2:**
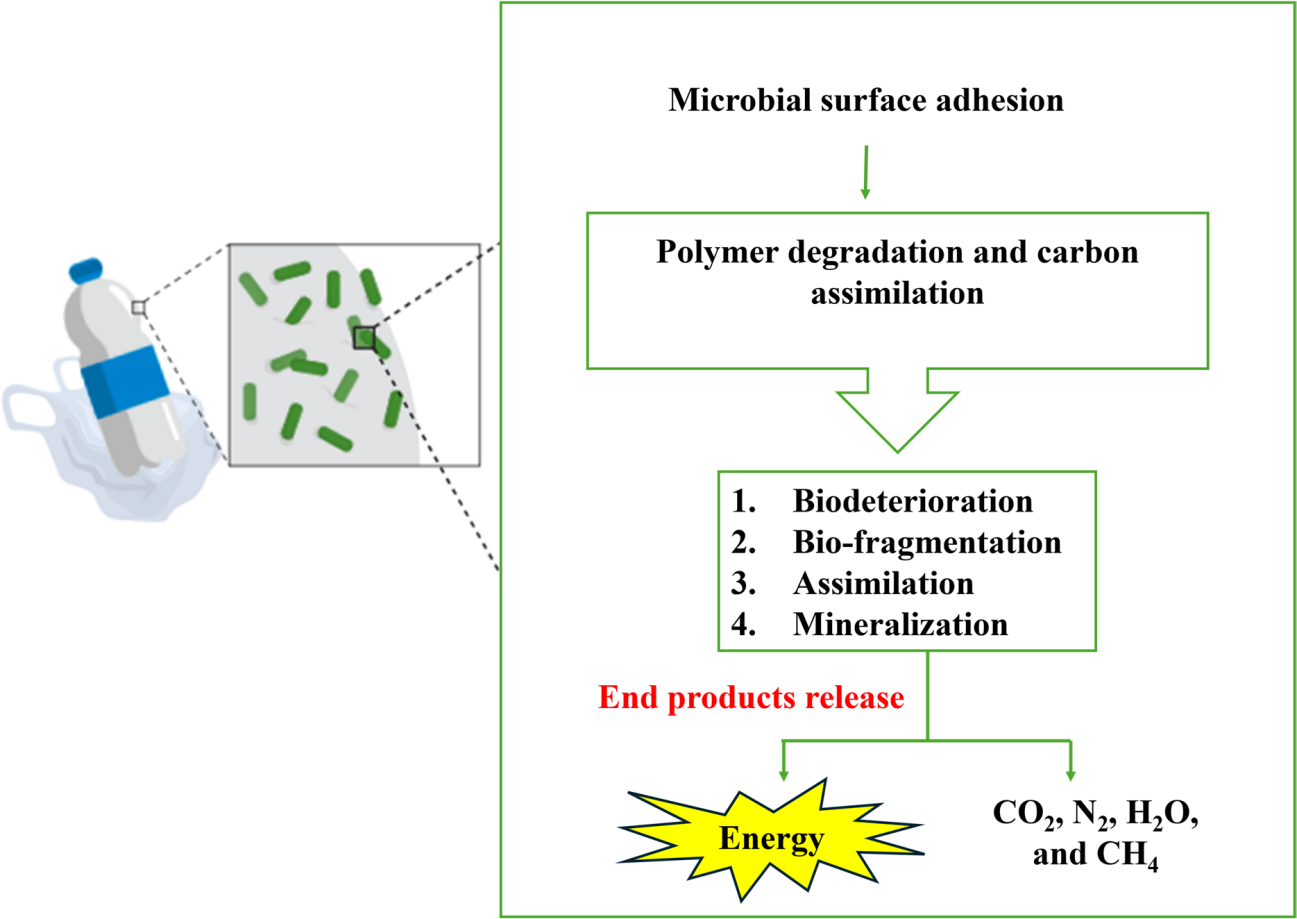
Schematic representation of the mechanism of plastic biodegradation showing key biological steps stating from bacterial adhesion to polymer surface till releasing of end products

Eventually, biomass is produced using this energy (Lucas et al. 2008; Kale et al. 2015). Mineralization is the process that results in inorganic species such as H_2_O, CH_4_,and CO_2_ (Gu 2003). Anaerobic or aerobic biodegradation may be used in this procedure (Malik et al. 2023). The absorption step entails the uptake of degradation products into the cell, where they undergo complete breakdown. To other microbes, secondary metabolites are transferred for further processing and utilization or expelled from cells. During the degradation of these metabolites, oxidation products (CO_2_, N_2_, H_2_O, and CH_4_) are generated (Krzan et al. 2006; Lucas et al. 2008). Figure 2 illustrated this mechanism.

### Factors affecting enzyme activity

In plastic-degrading bacteria, enzyme efficiency is shaped by a range of factors related to the properties of polymers and the conditions of exposure (Mohanan et al. 2020). These factors are grouped into three main categories: polymer-related factors, environmental factors and microbial- or enzyme- related factors.

#### Polymer-related factors

These factors affect the bacteria's ability to efficiently break down plastics and the overall rate of biodegradation. Key characteristics include physical and chemical traits, such as the presence of functional groups that enhance hydrophobicity (Wang et al. 2021a), structural complexity (Tokiwa et al. 2009), and the types of easily breakable bonds like amide and ester bonds. Additionally, the composition of molecules (Shams et al. 2020), the form of the polymer, its nature and physical appearance (e.g., pellet, films, powder) (Kawai et al. 1995), the density and molecular weight of the polymer (Tokiwa et al. 2009), the ratio of amorphous to crystalline regions (Wang et al. 2021b), and toughness all play significant roles, with softer degrading more quickly than harder ones (Swift 1993).

When polymers become less soluble, the ability of microbes to degrade them decreases. Because they are less soluble, plastics are less susceptible to microbial attack. Their cell membranes facilitate interactions with other microorganisms (Siracusa et al. 2008). Compared to crystalline polymers, amorphous polymers are more vulnerable to microbial enzyme assault. Accordingly, polymer degradation is reduced when crystallinity rises (Slor and Amir 2021).

#### Environmental factors

The surrounding environment plays a crucial role in shaping microbial activity and enzyme efficiency. In a hydrophobic plastic-contaminated environment, microbial activity can be reduced by preventing the absorption of water. Furthermore, crucial enzymes related to bacterial biodegradation are affected by exposure conditions, i.e., substrate concentration, pH, presence of inhibitors, temperature, synergy between microbes, salinity, oxygen level, light, and humidity.

The flexible amorphous portions of PET are more vulnerable to enzymatic and/or microbiological breakdown at temperatures greater than the glass transition temperatures (Tg). When polymer degradation occurred, the average chain length decreased because shorter chains were more motile, which led to a Tg decline (Odusanya et al. 2013). Additionally, greater temperatures were observed to enhance the polyesters' deterioration. Disinfected plastic garbage was also disinfected by higher temperatures (over 55°C) (Islam et al. 2019). Another aspect of the environment that might impact plastics' solubility and softening is pH. The capacity of acidophilic bacteria to break down synthetic polymers, however, has not received much attention. At the same time, some plastic items utilized in low pH bleaching methods exhibit reduced stability under acidic pH (Atanasova et al. 2021). Current understanding of alkaliphiles' capacity for degradation is scarce. According to Dussud et al. (2018), bacterial strains isolated from hyperalkaline water samples were able to destroy low-density polyethylene (LDPE). Reaction rate is associated with substrate concentration and surface area; when substrate concentration rises, the accessible surface increases as well, reaching a maximum rate (until enzyme saturation) (Chinaglia et al. 2018). Environmental factors such as temperature, salinity, oxygen concentration, and light limits play crucial roles in biofilm production (Dash et al. 2013; De Tender et al. 2015). Urbanek et al. (2018) suggest that increasing temperature and humidity may be essential for accelerating degradation rates.

#### Microbial or enzyme related factors

Additionally, Howard and McCarthy (2023) postulated that improving a bacterium's capacity to adhere to plastic and create a biofilm on it could optimize the enzyme's local concentration surrounding the target substrate, hence speeding up the pace at which plastic degrades overall. Furthermore, rather than the substrate, valeric acid, butyric acid, and hexanoic acid may interact as competitive inhibitors with the cutinase's substrate-binding site (Maeda et al. 2005). According to Jadhav et al. (2024), the elimination of CO_2_'s feedback inhibition resulted in an increase in bacterial proliferation and aptitude to degrade plastic. Siderophore chelators, such as bacillibactin, have been shown by Tkachuk and Zelena (Tkachuk and Zelena 2021) to slow down material degradation by altering surface hydrophobicity at high concentrations and preventing the formation of bacterial biofilms. Specialized microorganisms and secreted enzymes must work together to effectively break down plastics into substrates for bio-fermentation because they are extremely resistant (Blair et al. 2021). The activity of microbial consortia is significantly higher than that of individual strains (Cao et al. 2022). For improvement of plastic-degrading bacteria efficiency, it is fundamental to understand and optimize these factors, ultimately leading to improved waste management techniques.

## Genetics of bacterial plastic degradation

### Bacterial degradation of different synthetic polymers

Plastics are extensively utilized across various industries due to their remarkable adaptability, durability, and lightweight properties (Tamošaitienė et al. 2024). These polymers are formed through chemical bonds between repeated monomer units (Parisi et al. 2015). Notable examples include PE, commonly used in containers and packaging; PP, utilized in textiles and automotive parts; PET, employed in the manufacture of bottles and synthetic fibers; PVC, used in the construction industry (Akovali 2012; Singh and Demirsöz 2022); and nylon 6,6 microplastics (NMPs), utilized in both industrial and residential settings (Kale et al. 2015; Bilad et al. 2018). Low molecular weight polyethylene (LMWPE), linear low-density polyethylene (LLDPE), low-density polyethylene (LDPE), and high-density polyethylene (HDPE) are distinct classifications within the broader category of polyethylene, leading to variations in density and three-dimensional structure (Mohanan et al. 2020). While these plastics are essential to modern society, they face significant environmental challenges due to their resistance to natural degradation (Fotopoulou and Karapanagioti 2017). Current initiatives seek to address these issues by developing biodegradable polymers and enhancing recycling technologies, as well as promoting biodegradation of plastic through microbial and enzymatic methods (Alshehrei 2017).

Bacterial plastic degradation consists of complex steps involving enzymatic degradation, physiochemical modifications, and microbial interactions. Specific enzymes secreted by plastic-degrading bacteria i.e., PETase and mono(2-hydroxyethyl) terephthalate hydrolase (MHETase) in *I. sakaiensis*. played specific roles, with PETase converting PET into oligomers, including MHET as their primary component, and MHETase further hydrolyzing MHET to terephthalic acid (TPA) and ethylene glycol (EG) (Yoshida et al. 2021). Similarly, PU-degrading bacteria secrete lipase and esterases (Howard et al. 2001, 2007). For highly persistent plastics, oxidative pretreatments (UV irradiation, heating, and chemical oxidation) render the polymer susceptible to enzymes (Mohanan et al. 2020). Pre-treatment techniques that promote biodegradability by rupturing PE’s carbon chains have an impact on degradation efficiency. For instance, *Brevibacillus borstelensis* strains degraded up to 59.2% of thermo-degraded LDPE films, while marine bacteria like *Bacillus sphericus* and *Bacillus cereus* showed weight losses of 19% and 9%, respectively, in thermally treated plastics (Sudhakar et al. 2008; Abrusci et al. 2013). Co-culturing bacterial strains, such as *Rhodanobacter sp.*, *Rhodopseudomonas sp.*, and *Microbacterium sp.,* with *Bacillus aryabhattai* demonstrated superior degradation of PE mulching films (Wang et al. 2023). Additionally, *Pseudomonas knackmussii N1-2*, isolated from activated sludge, and *Bacillus vallismortis* from cow dung showed remarkable PE breakdown, with the latter achieving 75% LDPE degradation after 120 days (Park and Kim 2019; Hou et al. 2022). Significant degradation rates were also reported for *Enterobacter* and *Pseudomonas* strains from cow manure, achieving 64.25% and 63% degradation of LDPE and polypropylene, respectively (Skariyachan et al. 2021). Bacterial degradation of synthetic polymers is a critical research area in combating plastic pollution by various bacterial species isolated from different sites, as summarized in Table 1.

**Table 1:** Some types of bacteria and their efficiency in degrading different types of plastic isolated from different terrestrial and marine environments

Bacteria	Type of plastic	Source	Duration (days)	Degradation efficiency (%; indicator used)	Product of Degradation	Reference
*Terrestrial environment*						
*Brevibacillus brevis*	nylon 6, 6 microplastics	Soil	35	22% (weight loss %)	Amides, CO₂, oligomers	Tiwari et al. (2022)
*Brevibacillus borstelensis*	LDPE	Soil	90	30% (Weight loss%- FTIR)	CO₂, alcohols, ketones	Hadad et al. (2005)
*Streptococcus sp., Staphylococcus sp., Micrococcus sp., Moraxella sp., and Pseudomonas sp.*	PE	Soil	30	2.19–20.54% (weight loss %)	Not detected	Kathiresan (2003)
*Lysinibacillus sp.*	PE	Soil	26	9% (weight loss %)	Short-chain hydrocarbons	Jeon et al. (2021)
*Lysinibacillus sp.*	PP	Soil	26	4% (weight loss %)	Aldehydes, CO₂	Jeon et al. (2021)
*Serratia marcescens*	PE	The gut of waxworms larvae	60	3.57% (weight loss %)	Low MW hydrocarbons	Lou et al. (2022)
*Alcaligenes faecalis*	HDPE	Soil	40	5.8% (weight loss %)	CO₂, organic acids	Tareen et al. (2022)
*Alcaligenes faecalis*	LLDPE	Soil	40	3.5% (weight loss %)	CO₂, alcohols	Tareen et al. (2022)
*Bacillus siamensis*	LDPE	Soil	90	8.46% (weight loss %)	Alcohols, fatty acids	Maroof et al. (2021)
*Xanthomonas sp. HY-71*	Polyether-PU Foam	The gut of Japanese carpenter bee	14	10.95% (weight loss %)	Urethane oligomers, CO₂	Kim et al. (2022)
*Xanthomonas sp. HY-71*	Polyester-PU Foam	The gut of Japanese carpenter bee	14	23.95% (weight loss %)	Esters, CO₂	Kim et al. (2022)
*Xanthomonas sp. HY-71*	Acryl PU-Siegel	The gut of Japanese carpenter bee	3	88.12% (weight loss %)	Acrylic monomers	Kim et al. (2022)
*Enterobacter and Pseudomonas*	PP	Cow dung	160	63–64.25% (Weight loss % -XRD-FTIR)	CO₂, low MW compounds	Skariyachan et al. (2021)
*Acinetobacter sp.*	PS	The gut of *Tribolium castaneum* larvae	60	12.14% (weight loss %)	Styrene oligomers	Wang et al. (2020)
*Enterobacter and Pseudomonas*	LDPE	Cow dung	160	63–64.25% (weight loss% + FTIR + Tensile strength)	CO₂, ketones, acids	Skariyachan et al. (2021)
*Sporosarcina globispora*	PP	Mangrove sediments	40	11% (weight loss %)	CO₂, fatty acids	Auta et al. (2017)
*Bacillus paramycoides*	PE	Corals of Karimunjawa National Park	7	2.25% (weight loss %)	Not detected	Widyananto et al. (2021)
*Acinetobacter iwoffii*	LDPE	Soil	90	0.76% (weight loss %)	Not detected	Maroof et al. (2021)
*Pseudomonas aeruginosa*	LDPE	Soil	90	1.15% (weight loss %)	Alcohols, aldehydes	Maroof et al. (2021)
*Brevibacillus borstelensis, Bacillus cereus, Bacillus megaterium and Bacillus subtilis*	LDPE-Co	Soil	90	59% (CO₂ evolution + weight loss)	CO₂, alcohols, acids	Abrusci et al. (2013)
*Brevibacillus borstelensis, Bacillus cereus, Bacillus megaterium and Bacillus subtilis*	LDPE-Fe	Soil	90	9% (CO₂ evolution + weight loss)	CO₂, oligomers	Abrusci et al. (2013)
*Pseudomonas knackmussii N1–2*	PE	Sewage treatment plant	56	5.95% (weight loss %)	Not detected	Hou et al. (2022)
*Pseudomonas aeruginosa RD1-3*	PE	Sewage treatment plant	56	3.62% (weight loss %)	Not detected	Hou et al. (2022)
*Serratia sp.*	LDPE	Solid waste-dumping sites	150	40% (weight loss %)	CO₂, short alkanes	Nadeem et al. (2021)
*Stenotrophomonas sp.*	LDPE	Solid waste-dumping sites	150	40% (weight loss %)	Organic acids	Nadeem et al. (2021)
*Rhodococcus ruber*	PP	Mangrove sediments	40	6.4% (weight loss %)	Alkanes, ketones	Auta et al. (2017)
*Bacillus cereus*	PP	Mangrove sediments	40	12% (weight loss %)	CO₂, aldehydes	Auta et al. (2017)
*Gordonia sihwensis*	PS	Mangrove ecosystem	30	4.69–7.73% (weight loss %)	Styrene, benzoic acid	Liu et al. (2023)
*Bacillus vallismortis*	LDPE	Plastic contaminated with cow manure	120	75% (weight loss %)	Alkanes, CO₂	Gilani et al. (2023)
*Bacillus and Paenibacillus*	PE	Landfill site	60	14.7% (weight loss- FTIR)	CO₂, acids	Park and Kim (2019)
*Bacillus wiedmannii*	LDPE	Soil	90	5.39% (weight loss %)	Not detected	Maroof et al. (2021)
*Bacillus subtilis*	LDPE	Soil	90	3.75% (weight loss %)	Not detected	Maroof et al. (2021)
*Bacillus cereus*	LDPE	Soil	90	6.33% (weight loss %)	Not detected	Maroof et al. (2021)
*E. coli, Corynebacterium sp.*	Pink plastic	Waste disposal sites	28	45.7–46.4% (weight loss %)	Alkane/alkene	Javid et al. (2024)
*Pseudomonas sp.*	White plastic	Waste disposal sites	28	46.4% (weight loss %)	Alcohol	Javid et al. (2024)
*Bacillus sp.*	Black plastic	Waste disposal sites	28	47.4% (weight loss %)	Alcohol	Javid et al. (2024)
*Marine environment*						
*Marinobacter gudaonensis*	PET	Deep-sea sediments	30	1.2–1.3% (weight loss %)	Terephthalic acid (TPA), ethylene glycol	Zhao et al. (2023)
*Rhodococcus pyridinivorans P23*	PET	Marine sediment sample of the Pacific Ocean	35	4.28% (weight loss %)	TPA, monoesters	Guo et al. (2023)
*Bacillus sphericus*	HDPE and LDPE (thermally treated)	Shallow waters of the Indian Ocean	365	19% (weight loss + FTIR)	Alcohols, ketones	Sudhakar et al. (2008)
*Bacillus cereus*	HDPE and LDPE (thermally treated)	Shallow waters of the Indian Ocean	365	9% (weight loss + FTIR)	Aldehydes, fatty acids	Sudhakar et al. (2008)
*Bacillus sphericus*	LDPE (Untreated)	Sea water	365	10% (weight loss %)	Not detected	Khandare et al. (2021)
*Bacillus cereus*	LDPE (Untreated)	Sea water	365	5% (weight loss %)	Not detected	Khandare et al. (2021)
*Bacillus cereus*	LDPE	Plastic-polluted tropical coastal environment	42	4.13% (Weight loss%-FTIR)	Increase oxygen content, carbonyl, and vinyl	Jebashalomi et al. (2024)
*Bacillus cereus*	PS	Plastic-polluted tropical coastal environment	42	14.13% (weight loss%-FTIR)	Increase oxygen content, carbonyl, and vinyl	Jebashalomi et al. (2024)
Consortium of* Fulvimarina pelagi* + *Paracoccus halotolerans* + *Oceanicola granulosus*	PS	Marine plastic debris	45	18.9% (weight loss%-FTIR- hydrophobicity)	Oxidation, reduced hydrophobicity	Wang et al. (2025)
*Oceanimonas pelagia*	LDPE	Marine sediment, Taiwan’s Northern Coast				Yang et al. (2024
*Bacillus tropicus, Bacillus cereus, Stenotrophomonas acidaminiphila,* *Brucella pseudintermedia and Bacillus cereus*	PP	Thondi and Rameshwaram coast, India	28	51.5%, 47.5%, 33%, 28.5% and 35.5%, respectively (weight loss %-FTIR)	Alteration in carbonyl region of FTIR and produced CO₂ and water	Jeyavani et al. (2024)
*Pseudalkalibacillus sp.*	PE	The seawater, the nearshore of Liaodong Bay, Liaoning Province, China	60	6.3% (weight loss %-FTIR)	Carbonyl and hydroxyl groups could increase hydrophilicity and facilitate the adhesion on the surface	Meng et al. (2024)
*Micrococcus flavus*	HDPE	Coastal region of the Arabian Sea, Gujarat, India	30	1.8%(weight loss %-FTIR)	Oxidation of HDPE	Sardar (2025)
*Bacillus cereus*	PS microplastics	Mula River in Pune, India	30	20% (weight loss %-FTIR)	Appearance of hydroxyl and carbonyl functional groups and diminished aromatic C–H stretching, suggesting oxidative cleavage of the polymer	Kumari et al. (2025)
*Pseudomonas* sp.	PCL	Deep seawater	30	Clear-zone method	Not detected	Sekiguchi et al. (2011a, b)
*Arthrobacter* sp. and *Pseudomonas* sp.	HDPE	Marine ecosystem of Gulf of Mannar, India	30	12% and 15% respectively (weight loss %-FTIR)	Oxidation and chain modifications	Balasubramanian et al. (2010)
*Thalassospira xiamenensis*	PET	Pacific Ocean deep-sea sediments	30	1.3%–1.8%( weight loss%- SEM-UPLC-MS)	MHET-TPA	Zhao et al. (2023)
*Kocuria palustris*	PE	Arabian Sea seawater	30	1% (weight loss%	Increase keto carbonyl bond index, ester carbonyl bond index, and vinyl bond index	Harshvardhan and Jha (2013)
*Vibrio alginolyticus*, *Vibrio parahemolyticus*	PVA-LLDPE	Marine environments	450	20% (decrease tensile strength)	Not detected	Raghul et al. (2014)
*Marinobacter gudaonensis*	PET	Pacific Ocean deep-sea sediments	30	1.3%–1.8%( weight loss%- SEM-UPLC-MS)	MHET-TPA	Zhao et al. (2023)

With temperatures below 5°C, the majority of our world is perpetually cold and uninhabitable. The explanation for this is that seawater, primarily deep ocean, covers about 70% of the planet and two-thirds of it has a surprisingly consistent temperature of about 2°C (Russell 1990). Coastal tourism, fishing, maritime industries, and plastic production are the main contributors of synthetic plastic trash in marine habitats, and these activities have a direct effect on seas and oceans (Cole et al. 2011; Veiga et al. 2016). Additionally, plastic waste indirectly enters marine ecosystems through rivers and drainage systems, carrying pollutants from households and the cosmetic industry (Cole et al. 2011). This process often results in higher plastic concentrations near coasts and river estuaries. Plastic waste consists of primary and secondary microplastics in the marine environment (Veiga et al. 2016; Maes et al. 2017).

Each gram of wet marine sediment harboring hundreds of millions of bacterial cells indicates the vast number of microbes in marine environments (Harrison et al. 2011). Plastic debris, whether floating on the surface or submerged, is rapidly colonized by microorganisms, leading to the formation of biofilms within a few hours (De Tender et al. 2015; Eich et al. 2015; Rummel et al. 2017; Pauli et al. 2017). These microbial communities, known as the plastisphere, support various survival strategies such as horizontal gene transfer, nutrient accumulation, and defense against toxins (Zettler et al. 2013; Rummel et al. 2017). Furthermore, biofilms facilitate the adhesion of other species, including fungus, flagellates, and diatoms, which can result in biofouling (Pauli et al. 2017). This biofouling process adds weight to plastic waste, causing it to sink and potentially providing a habitat for invertebrates that feed on the biofilm (Reisser et al. 2014). Additionally, bio fouled plastics can transport invasive or non-native species to new locations, posing a threat to marine ecosystems (Urbanek et al. 2018). Research into the plastisphere and the microorganisms associated with marine plastic litter has gained attention due to the mounting threat of plastic pollution in the marine (Roager and Sonnenschein 2019). As illustrated in Table 1, numerous studies have focused on identifying plastic-degrading bacteria from marine environments that have the potential to break down various types of plastics.

Bacteria demonstrate an impressive ability to survive and adapt in harsh conditions such as cold environments, where they develop unique microbial traits (Urbanek et al. 2018). In these contexts, bacteria capable of breaking down plastic are garnering increased attention. Plastic waste in the oceans not only serves as a new substrate for benthic organisms but also releases dissolved organic carbon into seawater, which stimulates heterotrophic microbial activity (Pauli et al. 2017; Romera-Castillo et al. 2018). This adaptation to novel carbon sources has driven the evolution of cold-active enzymes in polar microorganisms (Rampelotto 2014). These enzymes hold significant potential for biotechnological applications and offer promising solutions to address plastic pollution (Rampelotto 2014).

### Key genes involved in bacterial plastic degradation

Numerous genes encoding enzymes and transport systems are involved in bacterial plastic degradation, which allows bacteria to use the plastic polymer as a carbon source and energy. Usually, these genes are involved in metabolic pathways that specifically target particular types of plastic polymers, as provided in Table 2.

**Table 2:** Genes involved in plastic degradation

Gene	Enzyme	Microorganism	Plastic	References
*est119*	Esterase	*Thermobifida alba*	PET	Hu et al. (2010)
*cut1*	Cutinase 1	*Thermobifida fusca *	PET	Herrero Acero et al. (2011)
*Tcur_1278*	Triacylglycerol lipase	*Thermomonospora curvata*	PET	Wei et al. (2014)
*Cut190*	Alpha/beta hydrolase family protein	*Saccharomonospora viridis*	PET	Kawai et al. (2014)
*cut2*	Cutinase	*Thermobifida fusca*	PET	Danso et al. (2018)
*ISF6_4831*	PETase	*Ideonella sakaiensis *	PET	Yoshida et al. (2016)
*NlhH/Aes*	Acetyl esterase/lipase	*Pseudomonas sp.*	PET	Roberts et al. (2020)
*nylC*	Endotype 6-aminohexanoat-oligomer hydrolase	*Agromyces sp. *	Nylon	Yasuhira et al. (2011)
*pueA*	Polyurethanase A	*P. chlororaphis*	PU	Howard et al. (2007)
*pueB*	Polyurethanase B	*P. chlororaphis*	PU	Howard et al. (2001)
*Oxr-1*	Oxidoreductase	*B. velezensis *	PU, PBAT	Gui et al. (2023)
*phaZ*	PHB depolymerase	*Schlegelella sp. *	PHB	Romen et al. (2004)
*styA, styB, styC and styD*	Main component of styrene monooxygenase, Styrene oxide isomerase and Phenylacetaldehyde dehydrogenase	*P. fluorescens *	styrene	Velasco et al. (1998)
*almA*	Baeyer–Villiger monooxygenase	*Acinetobacter baylyi *	PE	Yin et al. (2024)
*PsLAC*	Laccase	*Psychrobacter sp. *	PE	Zhang et al. (2022)
*alkB*	Alkane monooxygenase AlkB	*Paenibacillus sp.*	PE, LDPE	Bardají et al. (2019)

Several genes encode enzymes responsible for plastic degradation by microorganisms, each targeting specific types of synthetic polymers. *Est119* encodes an esterase that hydrolyzes polyester-based plastics (Hu et al. 2010). *Cut1, Cut2,* and *Cut190* encode cutinases, which break down PET and other PS materials by cleaving ester bonds (Herrero Acero et al. 2011; Kawai et al. 2014; Danso et al. 2018). *NylC* encodes a nylon hydrolase, facilitating the degradation of nylon-6 oligomers (Yasuhira et al. 2011). *PueA* and *PueB* encode polyurethane esterase enzymes that degrade polyurethane by breaking down ester linkages (Howard et al. 2001, 2007). *AlkB* encodes an alkane monooxygenase, a key enzyme in polyethylene degradation, initiating oxidation of the polymer backbone (Bardají et al. 2019). *Tcur_127*8 is associated with thermophilic plastic degradation, particularly for polyester and PET (Wei et al. 2014). *PhaZ* encodes a polyhydroxyalkanoate (PHA) depolymerase, responsible for degrading biodegradable plastics such as polyhydroxybutyrate (PHB) (Romen et al. 2004). The *StyA, StyB, StyC*, and *StyD* genes form a styrene degradation pathway, allowing bacteria to break down polystyrene into less harmful compounds (Velasco et al. 1998). An enzyme similar to alkane monooxygenases, which is encoded by *AlmA*, aids in the oxidation of long-chain hydrocarbons, such as PE (Yin et al. 2024). As well as, acetyl esterase/lipase superfamily Aes catalyze the hydrolysis of short-chain esters, including triacylglycerols and vinyl esters of PET (Roberts et al. 2020). Also, oxidoreductase *Oxr-1* is a key degradation enzyme toward marine PU and can degrade the biodegradable plastic polybutylene adipate terephthalate (PBAT) (Gui et al. 2023). A laccase, an essential enzyme to the oxidative degradation of several plastics i.e., PE and PS is encoded by *PsLAC* (Zhang et al. 2022). Furthermore, *ISF6_4831* encodes an enzyme similarly to PETase and has strong plastic-degrading activity (Yoshida et al. 2016). A wide range of microbial enzymes to degrade synthetic polymers are represented by these genes taken together, providing crucial information for bioremediation projects and environmentally friendly waste management plans. Also, recent advances in PU biodegradation highlight the role of esterases, proteases, lipases, and ureases produced by diverse bacterial and fungal taxa. These enzymes target ester and urethane linkages within PU (Antaliya et al. 2025).

In addition to these degradative enzymes, the efficient biodegradation needs transport systems and regulatory networks that enable the release of degradation products. For instance, the complementary relationship between PETase and MHETase is accompanied by specific transporters to do their function as *tphC* gene, which specifically recognizes and binds to TPA, transporting them to the vicinity of transporter channels in the cell membrane (Zhao et al. 2025). As well as, in PE-degrading bacteria, *alkS* gene regulates alkane oxidation and acts as a transcriptional regulator (Rojo 2009). Also in styrene-degrading bacteria, two-component regulatory systems coordinate the enzymatic degradation of the *styS* and *styR* genes, encoded for sensor kinase and response regulator respectively, which is a positive regulator of the styrene operon, inducing transcription upon styrene presence (Tischler 2015). These transporters and regulators play a critical role in connecting extracellular polymers to intracellular metabolism and energy.

Collectively, these genes encode various hydrolytic and oxidative enzymes participating in plastic degradation, highlighting their ecological role and their strong promise for biotechnological applications.

## Multi-omics technology's participation in bacterial plastic degradation

Computational biology and bioinformatics utilized databases, software, and web-based resources to analyze biological data (Bhatt et al. 2018). Genomic databases and molecular docking techniques are essential for studying bacteria that degrade plastic. Table 3 summarizes the key resources and websites that support this research. To gain an in-depth understanding of the molecular mechanisms of bioremediation of hazardous substances, computational biology techniques are crucial. These resources aid in predicting and understanding the genetic, molecular, and cellular components responsible for degradation (Kumar et al. 2016; Shukla et al. 2025).

**Table 3:** A list of essential tools and websites that can help in studying the plastic biodegradation process

Tools	Purposes	Links
*Genomic and protein databases*		
NCBI GenBank	Search bacterial genomes, enzymes, and degradation pathways	https://www.ncbi.nlm.nih.gov/genbank/
UniProt	Find protein sequences and functional annotations for plastic-degrading enzymes	https://www.uniprot.org/
Enzyme database (BRENDA)	Get biochemical data on enzymes involved in plastic degradation	https://www.brenda-enzymes.org/
KEGG (Kyoto Encyclopedia of genes and genomes)	Study metabolic pathways related to plastic biodegradation	https://www.genome.jp/kegg/
Modeling enzyme-plastic interactions and predicting bacterial functions		
AutoDock, PyMOL and chimera	Predicts enzyme binding to plastic molecules, visualizes enzyme-ligand interactions, and 3D modeling of protein-plastic interactions, respectively	http://autodock.scripps.edu/ https://pymol.org/2/ https://www.cgl.ucsf.edu/chimera/
SwissDock	Performs molecular docking for enzymes that degrade plastics	http://www.swissdock.ch/)
AlphaFold	Predicts protein structures, including plastic-degrading enzymes	https://www.alphafold.ebi.ac.uk/
*Microbial and Environmental Databases*		
PlasticDB	A database of microbial plastic degradation enzymes and pathways	https://plasticdb.bioinformatics.nl/
GOLD (Genomes online database)	Provides metagenomic data on plastic-degrading microbial communities	https://gold.jgi.doe.gov/
EzBioCloud	Identifies bacterial strains capable of plastic biodegradation	https://www.ezbiocloud.net/
BacDive (Bacterial diversity metadatabase)	Contains information on bacterial species, including plastic-degrading microbes	https://bacdive.dsmz.de/
Experimental and Biodegradation Testing Resources		
OECD biodegradation testing guidelines	Standard methods to assess plastic biodegradation by microbes	https://www.oecd.org/env/test-guidelines
Plastic biodegradation studies database (PlasticsEurope)	Research on biodegradable plastics and microbial degradation studies	https://www.plasticseurope.org/

**Fig. 3:**
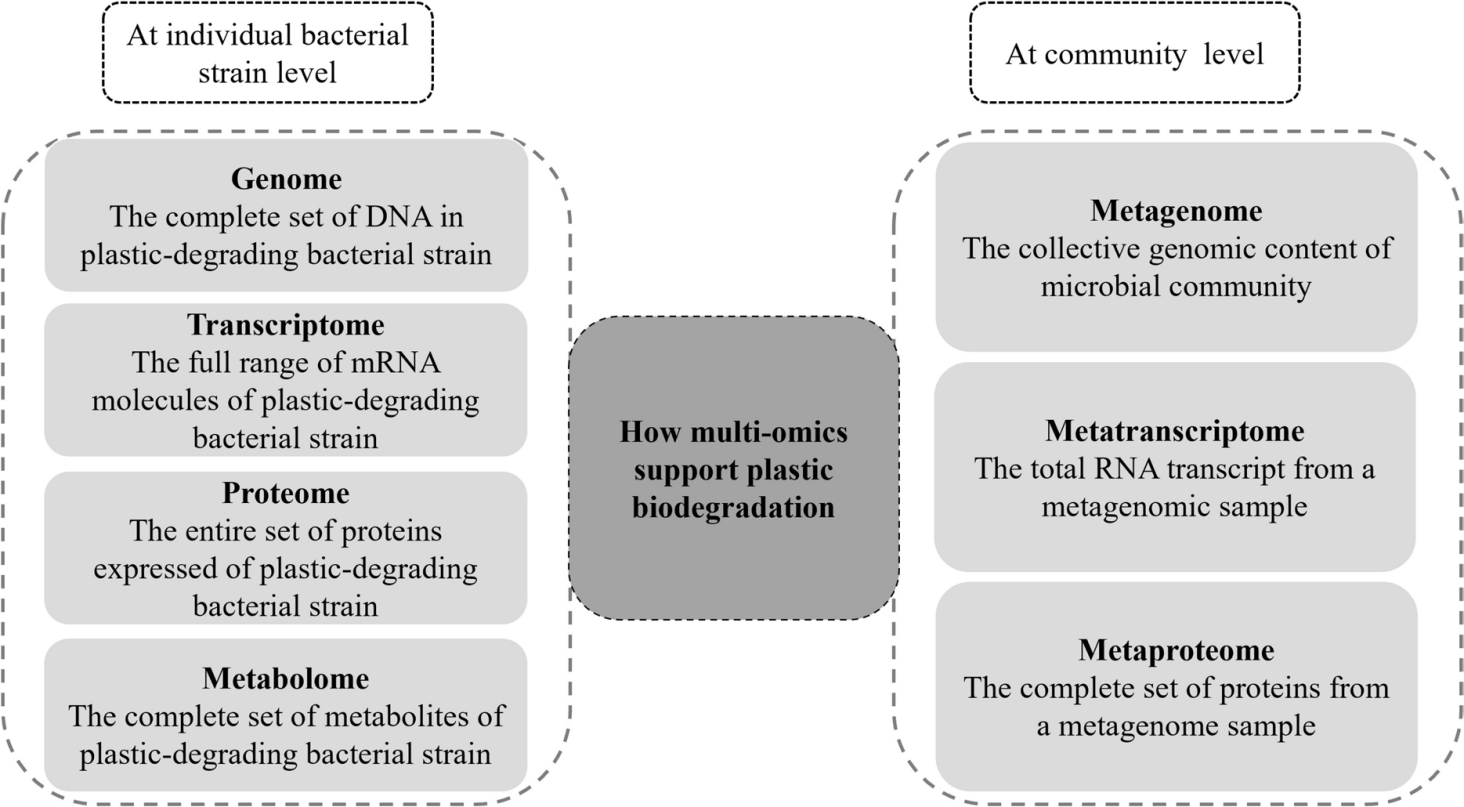
Multi-omics technology and comprehensive understanding of plastic-degrading bacteria at both individual and community levels

Multi-omics technology provides an integrated framework for elucidating plastic degradation mechanisms through linking different levels or different data types, as in Fig. 3. The key layers of information from the most basic to the most complex include the genome, transcriptome, proteome, metabolome, metagenome, metatranscriptome and metaproteome. These layers play a significant role in the degradation of pollutants (Halema et al. 2024) and in sustainability generally (Kaur et al. 2025; Kumar et al. 2025; Hassan et al. 2025). The integration of these multi-omics platforms enables high-resolution insights into microbial composition, metabolic pathways, and process resilience (Renganathan et al. 2025). Additionally, integrating multi-omics data provides a systems-level understanding of microbe interactions, microbial community dynamics, and the complex biological processes involved in bioremediation (Alidoosti et al. 2024). This enables predictive modeling and rational design of robust, efficient microbial inoculants and bioremediation strategies (Jain et al. 2024).

Representative case studies for genomics, metagenomic, transcriptomic, proteomic and metabolomic techniques have been successfully applied to detect plastic-degrading enxymes, reconstruct metabolic pathways, and verify active biodegradation in both isolated and natural plastisphere communities.

Whole genome sequencing (WGS) is an advanced technique used to sequence an organism’s entire DNA (Zhao and Grant 2011). This method has been instrumental in identifying microbes with the genetic potential for bioremediation of environmental pollution (Elarabi et al. 2023; El-Beltagi et al. 2024; Abdelhadi et al. 2024; Halema et al. 2025). According to Furlan et al. (2021),WGS enables the identification of essential genes that encode enzymes, specifically alkane hydroxylases, which are responsible for plastic biodegradation. This study focuses on the genome of the first strain of *Paenibacillus* sp. strain DK1, known for its ability to degrade PE. In addition, newly identified biodegradative enzymes for different plastic polymers were used to predict novel plastic-degrading enzymes from *Rhodococcus* genomes. Notably, Zampolli et al. ( 2024) provides the first evidence of the biodegradative importance of bacteria within the *Rhodococcus* genus, linking these metabolic capabilities to their genomic characteristics through the analysis of completely sequenced genomes and the genetic factors responsible for the biodegradation of PCL. As well as Shao et al. (2019), they present the complete genome sequence of *Streptomyces albogriseolus* LBX-2 and participate in understanding the genetic capacity and potential enzymatic pathways involved in PE degradation. Also, there are numerous genomic studies that help us to understand the genetic scenario behind the degradation of plastic as listed in Table 4.

**Table 4:** Some genomic studies responsible for understanding the plastic degrading bacteria

Bacterial species	Plastic type	Genome size (Mb)	Key enzymes / Genes	Sequencing platform	Reference
*Ideonella sp. Strain A 288*	PET	~ 6.9	PETase, MHETase	Illumina NextSeq	Braun et al. (2017)
*Paenibacillus aquistagni* strain DK1	PE	~ 5.4	laccases, manganese peroxidases, and alkane hydroxylases	Illumina MiSeq	Furlan et al. (2021)
*Streptomyces Albogriseolus LBX-2*	PE	~ 7.5	Oxygenases and other alkane	PacBio	Shao et al. (2019)
*Bacillus toyonensis *Cbmb3	PVC	~ 5.8	Dehydrogenases, peroxidase and dioxygenase	Oxford NanoDrop	Wang et al. (2024)
*Pseudomonas chengduensis* BC1815	PET	~ 5.8	Esterase, lipase and α/β hydrolase	hybrid sequencing strategy (Illumina and Oxford Nanopore)	Shi et al. (2023)
*Bacillus paralicheniformis* G1	PS	~ 4.3	Monooxygenase, dioxygenase, peroxidase, esterase and hydrolase	Illumina Hi-seq 4000	Kumar et al. (2021)
*Bacillus velezensis* GUIA	PUR	~ 3.9	Oxidoreductase, lipase and protease	hybrid sequencing strategy (Illumina and Oxford Nanopore)	Gui et al. (2023)
*Stenotrophomonas acidaminiphila* BDBP 071	Dibutyl phthalate	~ 3.9	Monoester hydrolase	Illumina Hiseq	Zhang et al. (2023)
*Rhodococcus erythropolis* D4 and *Rhodococcus opacus* R7	PCL	~ 6.4	Esterases, depolymerases, carboxylic-ester hydrolases lipases and cutinases	Illumina MiSeq	Zampolli et al. (2024)
*Stenotrophomonasindicatrix strain DAI2m/c*	PS	~ 4.7	Enzymes for a complete styrene degradation pathway	hybrid sequencing strategy (Illumina and Oxford Nanopore)	Zarra et al. (2025)

It's worth noting that metagenomic studies of plastic-contaminated environments provide a more comprehensive understanding of plastic degradation microorganisms. Metagenomic studies provide the understanding of plastic biodegradation by investigating the real-world microbial ecosystems that naturally interact with plastic waste (Kumar et al. 2024). Discoveries of metagenomics from marine environments, landfills, and wastewater reveal the global distribution of plastic-degrading potential (Kim et al. 2022). In addition to enhancing our understanding of enzymatic PET degradation, the metagenomic findings reported by Jahanshahi et al. (2025) provide a novel approach to identify and characterize possible biocatalysts. The stability and catalytic potential of these enzymes were also assessed using structure-based modeling and activity prediction algorithms, which were validated through laboratory tests conducted in specific environmental conditions. Furthermore, researchers identified a diverse and previously unknown bacterial community that has adapted to the plastic-polluted mangrove ecosystem through metagenomic sequencing (Pawano et al. 2024). Frey et al. (2024) explored the potential of alpine soil plastisphere reservoirs for novel degrading enzymes. A comprehensive metagenomic analysis can also illuminate the fitness, adaptability, and survivability of the core microbial population. This approach will facilitate the design of the microbial communities and their metabolic processes, ultimately enhancing degradation efficiency (Purohit et al. 2020). Additionally, Jahanshahi et al. (2023) conducted an extensive analysis of plastizymes.

Metagenome fills critical gaps, which WGS can’t do that by enabling the analysis of the whole community (cultured or uncultured) directly from environments and enabling more accurate metabolic interpretation. On the other hand, WGS is limited to cultured organisms and can’t capture microbial interactions (Liu et al. 2022). The researchers compiled a list of genes and proteins linked to plastic degradation using metagenomic data from various ecological environments. This catalog facilitates the study of enzymes’ evolutionary histories, functional diversity, and taxonomic distribution of these enzymes. The study mapping the occurrence of a wide variety of plastizymes, i.e., cutinases, lipases, esterases, and oxidoreductases, across microbial taxa, aims to identify both established and novel candidates involved in the plastic degradation (Jahanshahi et al. 2023). This work serves as a valuable resource for understanding the inherent capability of microbial communities to decompose materials, establishing a foundation for biotechnological applications such as designing microbial consortia and engineering enzymes to improve the handling of plastic waste management. This research enhances our ability to identify, compare, and utilize plastic-degrading genes and organisms globally. Furthermore, de Vogel et al. (2021) examined the diversity and evolutionary conservation of these enzymes across various marine bacterial species, employing comparative genomics to pinpoint key genes and metabolic pathways that facilitate the biodegradation of PHAs. To determine which genes are actively expressed during the biodegradation of PET and PE films by the marine bacteria *Rhodococcus opacus* R7 and *Bacillus* sp. AIIW2, the researchers conducted transcriptomic analysis at the RNA level (Kumari et al. 2021; Zampolli et al. 2021). Additionally, during the biofilm’s formation, changes in gene expression were also tracked using metatranscriptomic analysis, which identified enzymes and metabolic pathways potentially involved in HDPE degradation (MacLean et al. 2024).

Proteomics and metabolomics enhance our understanding of microbial responses to plastic pollution, facilitating the progress toward sustainable and more effective bioremediation techniques. Proteomics provide essential insights into the life cycle, regulation, and post-translational modifications of proteins induced under specific conditions (Cho 2007; Aslam et al. 2017). The ability of *Streptosmyses* sp. PU10's to not only degrade PU but also redirect its degradation of intermediates into secondary metabolite pathways illustrates its potential to transform byproducts into beneficial substances (Pantelic et al. 2024). To fully understand how *Ideonella sakaiensis* effectively degrades and assimilates PET, a thorough examination of the proteins involved in PET degradation is necessary (Poulsen and Nielsen 2023). Using proteomic analysis, researchers evaluated the expression of plastizymes in isolated strains exposed to various plastics i.e., PE and PET (Rüthi et al. 2023). The study found that these microbes not only survive harsh conditions but also upregulate specific proteins that facilitate the low-temperature degradation of plastic.

It is worth noting that integrated multi-omics techniques provide higher-resolution insight than single-omics studies by linking genetic potential to active function. For instance, Oberbeckmann et al. (2021) conducted metagenomic and proteomic analyses, revealing that microbial communities consistently express stress-response proteins and plastizymes, regardless of the type of plastic involved. This suggests that ambient factors and microbial interactions have a greater impact on biofilm functionality than the inherent properties of the plastic (Wright et al. 2020). Furthermore, advanced sequencing technologies i.e., metagenomics, metatranscriptomics, and metaproteomic, can uncover new enzyme activities and biodegradation processes, as well as identify and characterize microbial communities and their functional genes. These methods present promising opportunities for developing sustainable plastic waste management strategies (Viljakainen and Hug 2021).

## Investigating the role of biotechnological research in reducing plastic pollution

Research in synthetic biology and biotechnology offers more effective strategies for reducing plastic waste. Microbial cells can be genetically modified to enhance the plastic degradation in various environments. Genetic engineering includes a range of techniques, such as gene cloning, recombinant DNA technology, genetic modification, and genetic manipulation (Jaiswal and Shukla 2020).While these methods are still in their early stages, recent advancements in genetic engineering techniques like CRISPR/Cas9, TALENs, and zinc finger proteins have facilitated genetic experiments (Jaiswal and Shukla 2020; Shamshirgaran et al. 2022; Rezaei et al. 2024). It can perform various modifications with high precision, including knocking out unwanted genes, inserting entirely new ones, replacing existing ones with improved versions, or even fine-tuning gene regulation (Rafeeq et al. 2023). In particular, CRISPR based platform exemplified by PlastiCRISPR provides a transformative solution to plastic pollution by harnessing genome editing to enhance microbial plastic degradation and recycling capabilities (Palit et al. 2025).

Strategies such as designing artificial microbial consortia, metabolic engineering, and enzymatic engineering aim to optimize plastic degradation by redirecting bacterial metabolism and enhancing the catalytic efficiency and stability of plastizymes (Qi et al. 2021; Gao et al. 2024). These methods seek to improve plastic degradation by rerouting and optimizing bacterial metabolism, increasing the catalytic efficiency and stability of plastizymes, and integrating multiple microbes with complementary capabilities to maximize efficiency. In Table 5, these strategies are summarized.

**Table 5:** Some examples of biotechnological strategies to mitigate plastic pollution

Strategy	Target	Tools	Examples	References
Metabolic engineering	Pathway optimization	Gene knock in / out	Heterogenous expression of the PETase gene from *Ideonella sakaiensis* into *E. coli*, allowing the engineered strain to produce the PETase enzyme	Benavides Fernández et al. 2022; Effendi et al. 2024
			Metabolic engineering of *Pseudomonas putida* to express PU-degrading enzymes facilitates depolymerization of PU into its constituent chemicals, such as 1,4-butanediol and ethylene glycol	Ackermann et al. 2021
Enzyme engineering	Enzyme performance	Direct evolution, site directed mutagenesis	Structure guided enzyme engineering expands the active site aperture of PETase in *I. sakaiensis*, *Pseudomonas aestusnigri* hydrolase, and Cut190 in *Thermobifida cellulosilytica*,improving accessibility to polymeric PET substrates	Herrero Acero et al. 2011; Son et al. 2019; Rezaei et al. 2024
			Site-directed mutagenesis introducing an additional hydrogen bond to PETase in *I. sakaiensis,* enhancing enzyme stability and catalytic efficiency toward PET	Rezaei et al. 2024
			Replacement of the calcium binding site from *Thermobifida fusca* cutinase by a disulfide bridge increasing thermal stability of this enzyme	Then et al. 2016
			Engineering of *pelB* signal peptide in *E.coli*, improves extracellular production of IsPETase and increasing effective PET hydrolysis	(Shi et al. 2021)
Synthetic consortia	Synergistic effect	Microbial consortia design	A synthetic microbial consortium combining *E. coli* expressing PETase with *Pseudomonas putida* engineered for PU degradation, enables degradation of both PET and PU simultaneously through complementary metabolic functions	(Adamu et al. 2023)
			A synthetic microbial consortium of *Arthrobacter* sp. and *Streptomyces* sp. enhances degradation of PE film through improved biofilm formation	(Han et al. 2020)
			An artificial bacterial consortium dominated by *Rhodococcus*exploits a competitive plastisphere species to enhance plastic surface degradation	(Putcha and Kitagawa 2024)
			Environmental consortium containing *Pseudomonas* and *Bacillus* species synergistically degrades PET plastic through combined hydrolytic and metabolic pathways	(Roberts et al. 2020)
			Mixed microbial consortia show effective plastic degradation with various polymer types and formats (LDPE, LLDPE, and PET plastics),indicating broad substrate versatility	(Salinas et al. 2024)
			long-term experimental evolution over 40 selection cycles, using PE as the sole carbon source, to enrich microbial populations capable of more efficient PE degradation	(Li et al. 2025)

Despite their promise, synthetic biology particularly the environmental application of genetically engineered microorganisms, raises biosafety and regulatory concerns, including unintended environmental impacts, horizontal gene transfer, and persistence in the natural ecosystem. Furthermore, even if the cost of DNA synthesis has declined, making high-throughput genetic engineering more affordable, ethical, legal, regulatory, biosafety, and biosecurity issues still prevent these technologies from being used more widely. Regulatory frameworks should evolve to keep pace with technological advances to ensure safe and responsible application (Lea-Smith et al. 2025).

## Future prospective

Despite substantial efforts to identify the microbes responsible for plastic degradation and its upcycling, the industrial application of these technologies has not met expectations (Lee et al. 2023). Multi-omics approaches are essential for gaining a deeper understanding of microbial activities and their degradation potential. By employing these innovative methods, researchers can enhance their knowledge of the metabolic capabilities, ecological roles, and adaptive strategies of microbes linked to marine plastics (Messer et al. 2024).

**Fig. 4:**
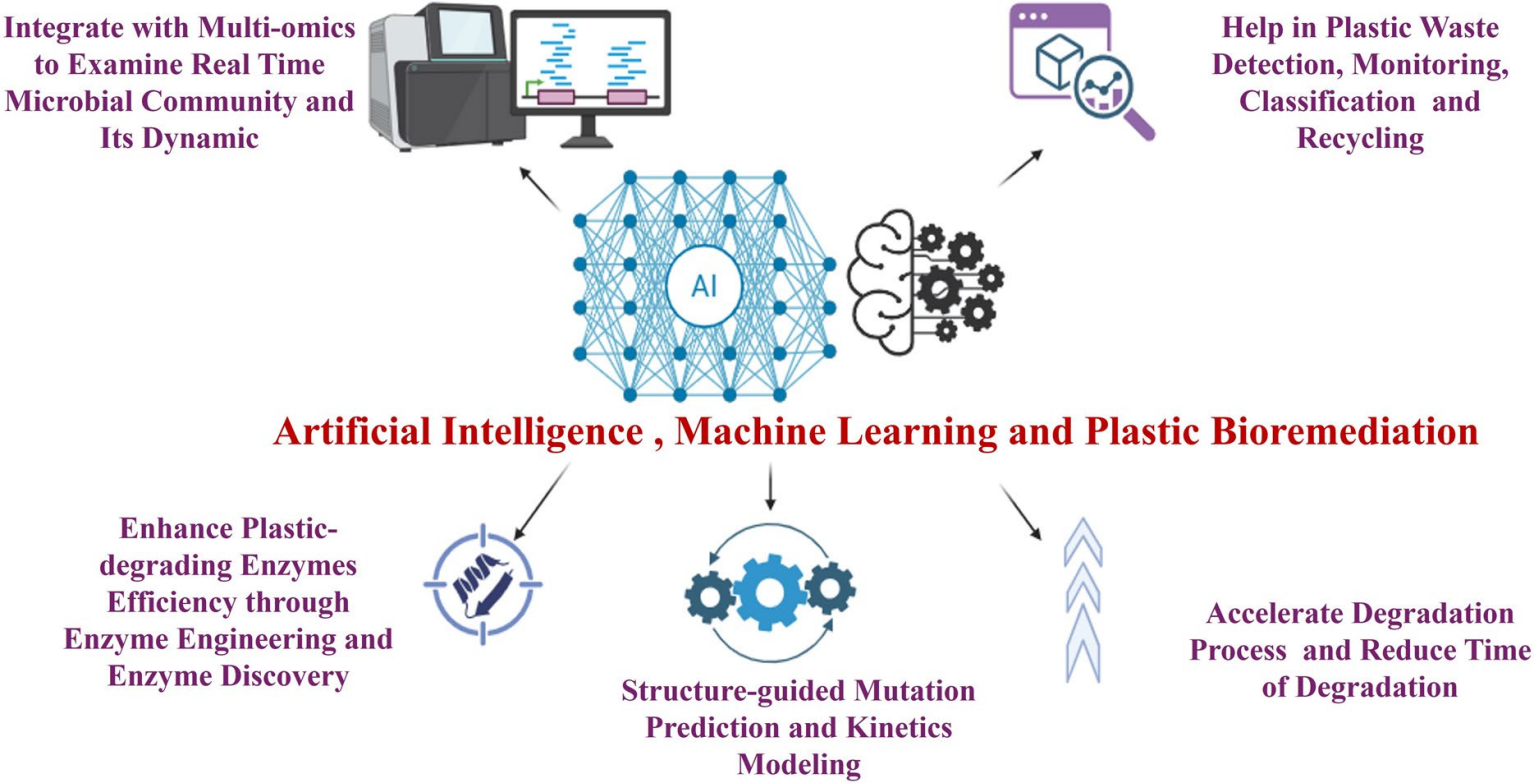
Artificial intelligence (AI) and machine learning (ML) applications in plastic bioremediation through predictive modeling

Artificial intelligence (AI) plays a crucial role in addressing environmental pollution, pollution control, and accelerating the development of sustainable techniques as proposed in Fig. 4. Recent advancements in AI algorithms are enabling modern methods for detecting, separating, and estimating the decomposition rates of a range of plastic waste materials. These algorithms significantly improve recycling systems by streamlining the improved separation and recovery of recyclable plastics (Maraveas et al. 2023; Gopalan and Ramakrishnan 2024). AI reduces the number of experimental cycles needed to identify specific enzymes, and its application in direct evolution is becoming increasingly popular. By utilizing AI to analyze characteristics of variations discovered during screening, researchers can select sequences more likely to exhibit desired functions (Lee et al. 2023). Machine learning (ML) algorithms can model the breakdown of plastic waste in landfills, develop biodegradable plastics with enhanced degradation properties, identify microplastics in environmental contexts, and assess the environmental impacts of degradation (Maraveas et al. 2023; Gopalan and Ramakrishnan 2024). Moreover, by analyzing extensive datasets of enzyme structures and functional properties, AI can predict and suggest optimal sequence or structural modifications which enhance enzyme function (through predicting the active site mutations), thereby accelerating degradation rates (through modeling the reaction kinetics of depolymerization) (Jang et al. 2022). It is worth noting that AI enhances the detection of microplastics by enabling automated image and spectral analysis, improving the classification of these polymers, and supporting advanced monitoring across different ecosystems (Zhao et al. 2024; Gaur et al. 2026). As well as, a three-dimensional (3D) self-supervised convolutional neural network (CNN) called MutCompute, ML algorithm, was used to engineer PETase enzyme called FAST-PETase to work faster under broader conditions and efficiently degrade PET within a few days. Also, researchers used the degraded plastic to produce new plastic, demonstrating a closed-loop recycling system (Lu et al. 2022). Consequently, the AI guided enzyme engineering represents a powerful technology further economically and scalability assessments are needed to validate their feasibility.

Despite the potential of AI and multi-omics approaches in plastic biodegradation, several limitations remain. These limitations can be concluded in ethical concerns about environmental justice, socioeconomic repercussions, and unexpected consequences, requiring vast amounts of high-quality data to train and evaluate predictive models, model biases, and limitations associated with implementation (Blessing and Olateru 2025). Also, multi-omics has numerous challenges i.e. vast amount and complexity of data and data analysis, data filtering and cleaning issues, curation, imputation, transformation, normalization, and scaling, data integration, fusion, clustering, visualization, and functional characterization (Gruszecka-Kosowska et al. 2022).

Importantly, Renganathan (2025) examined the integration of multi-omics platforms with AI-driven biosensing for real-time microbial community profiling and evaluated the structure and functional dynamics of microbial consortia in both aerobic and anaerobic treatment systems. The integration of multi-omics and AI should be prioritized to enable predictive functional modelling, reduce model bias, and facilitate data analysis. Also, the development of such technologies and regulatory and ethical frameworks will be critical for the safe application of them in plastic biodegradation.

## Conclusion

Plastic biodegradation is a promising and eco-friendly approach to tackling the growing global plastic waste problems. Various bacterial species have shown the ability to partially degrade synthetic polymers through complex biochemical processes and specialized enzymes. However, challenges remain regarding the efficiency, specificity, and scalability of these natural mechanisms. Recent advances in multi-omics have provided shed lights on the intricate metabolic and regulatory networks that drive bacterial plastic degradation, providing a system-level understanding of microbial responses to synthetic polymers. Additionally, biotechnological developments are paving the way for more effective and controllable plastic biodegradation methods. Despite ongoing challenges, particularly in environmental applications and large-scale depolymerization, the combination of molecular biology, systems science, and bioengineering offers a robust toolkit for converting plastic waste into valuable bioproducts or safe end products. This review highlights a comprehensive and integrative viewpoint of bacterial plastic biodegradation through connecting biodegradation with multi-omics techniques. Continued research, development, and interdisciplinary collaboration will be crucial for realizing the full potential of bacterial plastic degradation in a circular and sustainable bioeconomy.

## Data Availability

No datasets were generated or analysed during the current study.

## Abbreviations

AI: Artificial intelligence
PE: Polyethylene
PS: Polystyrene
PP: Polypropylene
PVC: Polyvinyl chloride
PET: Polyethylene terephthalate
PUR: Polyurethane
PCL: Polycaprolactone
Tg: Glass Transition temperatures
LDPE: Low-density polyethylene
NMPs: Nylon 6,6 microplastics
LMWPE: Low molecular weight polyethylene
LLDPE: Linear low-density polyethylene
HDPE: High-density polyethylene
PHA: Polyhydroxyalkanoate
PHB: Polyhydroxybutyrate
WGS: Whole genome sequencing
3D: Three-dimensional
CNN: Self-supervised convolutional neural network
